# MicroRNA Expression Patterns of CD8+ T Cells in Acute and Chronic Brucellosis

**DOI:** 10.1371/journal.pone.0165138

**Published:** 2016-11-08

**Authors:** Ferah Budak, S. Haldun Bal, Gulcin Tezcan, Furkan Guvenc, E. Halis Akalin, Guher Goral, Gunnur Deniz, H. Barbaros Oral

**Affiliations:** 1 Department of Immunology, Faculty of Medicine, Uludag University, Bursa, Turkey; 2 Department of Medical Biology, Faculty of Medicine, Uludag University, Bursa, Turkey; 3 Department of Molecular Genetics, Faculty of Medicine, University of Toronto, Toronto, Canada; 4 Department of Clinical Microbiology and Infection Diseases, Faculty of Medicine, Uludag University, Bursa, Turkey; 5 Department of Medical Microbiology, Faculty of Medicine, Uludag University, Bursa, Turkey; 6 Department of Immunology, Aziz Sancar Institute of Experimental Medicine, Istanbul University, Istanbul, Turkey; East Carolina University Brody School of Medicine, UNITED STATES

## Abstract

Although our knowledge about *Brucella* virulence factors and the host response increase rapidly, the mechanisms of immune evasion by the pathogen and causes of chronic disease are still unknown. Here, we aimed to investigate the immunological factors which belong to CD8+ T cells and their roles in the transition of brucellosis from acute to chronic infection. Using miRNA microarray, more than 2000 miRNAs were screened in CD8+ T cells of patients with acute or chronic brucellosis and healthy controls that were sorted from peripheral blood with flow cytometry and validated through qRT-PCR. Findings were evaluated using GeneSpring GX (Agilent) 13.0 software and KEGG pathway analysis. Expression of two miRNAs were determined to display a significant fold change in chronic group when compared with acute or control groups. Both miRNAs (miR-126-5p and miR-4753-3p) were decreased (p <0.05 or fold change > 2). These miRNAs have the potential to be the regulators of CD8+ T cell-related marker genes for chronic brucellosis infections. The differentially expressed miRNAs and their predicted target genes are involved in MAPK signaling pathway, cytokine-cytokine receptor interactions, endocytosis, regulation of actin cytoskeleton, and focal adhesion indicating their potential roles in chronic brucellosis and its progression. It is the first study of miRNA expression analysis of human CD8+ T cells to clarify the mechanism of inveteracy in brucellosis.

## Introduction

Brucellosis is a zoonotic bacterial disease caused by bacteria of the genus *Brucella*, which are able to establish long-term infections in their hosts [1, 2]. It remains endemic in many developing countries, causing significant economic loss and poses public health risks. Brucellosis bacteria are facultative intracellular Gram-negative bacteria that are 0.6–1.5 μm long, do not form spores and establish infection in macrophages [3]. According to host specificity there are 10 species of *Brucella*: *B*. *melitensis* (goat and sheep), *B*. *abortus* (cattle), *B*. *suis* (swine), *B*. *canis* (dogs), *B*. *ovis* (sheep), *B*. *neotomae* (desertmice), *B*. *cetacea* (cetacean), *B*. *pinnipedia* (seal), *B*. *microti* (voles), and *B*. *inopinata* (unknown) [4]. However, among recognized *Brucella* species, *B*. *melitensis*, *B*. *abortus*, *B*. *suis*, *B*. *canis* and more recently *Brucella pinnipedialis* and *Brucella ceti* have been associated with human disease [5]. *B*. *melitensis* accounts for the majority of the brucellosis cases in humans in Turkey [6]. Humans can acquire the bacteria through various ways including direct contact with diseased animals or by consumption of *Brucella* contaminated animal products. Human brucellosis is a disease presented either as generalized febrile illness, without an impact on organ systems, or as a focal disease, when one or more organs are involved [7]. Osteoarticular brucellosis is the is the most common focal form, with a frequency of 19–69% of the total number of brucellosis patients [8, 9]. Symptoms such as fever, fatigue, loss of appetite, headache, back ache, weight loss, myalgia and arthralgia are observed in acute brucellosis [10]. The acute phase of brucellosis follows the eradication of the bacteria by the immune system. Failure to clear the brucellosis infection in the acute form leads to transition into the chronic form defined by mild fever, sweating, weight loss and localized infections which resemble chronic fatigue syndrome. Chronic infection is seen in about 10–30% of the patients despite early diagnosis and treatment [11, 12]. The diagnosis of chronicity is mainly based on clinical symptoms and findings together with the presence of high IgG titers determined by serological tests [13]. However, serological assays maintain low specificity when it comes to diagnosis of brucellosis as IgG titers may remain positive for years following the successful resolution of symptoms [11, 14]. These facts suggest that good markers for clinical prediction, diagnosis and follow-up of brucellosis are needed to provide effective and accurate treatment regiments. Additionally, mechanisms leading to chronicity are not completely established. Therefore, understanding of mechanisms involved in evasion from host immune responses will provide valuable information about the pathogenesis of brucellosis and shed light on the development of new treatment strategies for the treatment of brucellosis and the prevention of transition to chronic form of the disease.

MicroRNA (miRNA) is a small RNA set with approximate length of about 22 nt. miRNA forms about 1–4% of the RNA in human genome. They target messenger RNA in a sequence specific fashion to carry out important regulatory functions. A single miRNA can regulate about 200 messenger RNA functions. It becomes active by breaking up the target messenger RNAs or by suppressing its translation depending on the extent of sequence specificity between the miRNA and mRNA [15, 16]. miRNAs take part in many functional developments and diseases such as hematopoesis regulation, cellular proliferation, cell differentiation, organogenesis, apoptosis, cancer development, infection development and heart disease [17–23].

Activated CD8+ CTLs contribute to protective immunity against Brucella through Fas- or perforin-mediated cytotoxicity and IFN-γ secretion [24–26]. In a murine brucellosis model, CTLs were found to increase during the chronic stage of infection [27]. In a similar manner, human clinical studies have also demonstrated increased numbers of CTLs in the peripheral blood of patients with chronic/relapsing brucellosis [28–30].

The objectives of our study were to investigate the miRNA expression changes of the CD8+ T cells that play important roles in the clearance and establishment of chronicity of Brucella infections. To find out which of the miRNAs that affect the transition to chronicity, the expression changes of the miRNAs involved in immune responses of CD8+ T cells were examined in acute and chronic brucellosis infections as well as healthy control groups.

## Materials and Methods

### Ethics Statement

This study protocol was approved by the Committee on the Ethics of the University of Uludag, Faculty of Medicine (Permit number: 2010-6/2). All participants gave written informed consent for inclusion.

### Inclusion of Participants

Seven patients with acute (3 female, 4male) and 8 chronic (6 female, 2 male total) cases of brucellosis with bone and joint involvement who have applied to the Uludag University Faculty of Medicine Clinical Bacteriology and Infection Diseases Department and/or patients who are already being followed along with 8 healthy controls (4 female, 4 male) were included in the study. The cases were classified according to the onset time of the symptoms as acute (0–2 months) and chronic (>12 months) [31, 32]. Brucellosis diagnosis was made with the existence of one or more constitutional symptoms such as fever, perspiration, fatigue and the standard tube agglutination test titre being ≥1/160 [33]. Patients were treated with Doxycyline 100 mg PO twice daily and Rifampicin 600–900 mg PO daily for 45 days [34].

### Fluorescence activated cell sorting (FACS) of CD8+ T cells and RNA isolation

Peripheral blood mononuclear cells (PBMCs) were separated from the 20 ml blood samples obtained from the patients in acute, chronic and healthy control groups via intensity gradient centrifuge method with Ficoll (Biochrom, Germany). PBMC fraction of patient whole blood was stained using CD4-FITC, CD8 PE, CD3 PerCP (Beckton Dickinson (BD), Biosciences, USA) antibodies to distinguish the CD8+ T cells for FACS. Antibodies were supplied by BD Biosciences, Inc. (USA) and used at the minimal saturating concentration, determined by titration prior to the experiment. PBMCs were stained with the antibody combination for 20 minutes at room temperature, washed twice and resuspended in PBS supplemented with 1% FCS. CD8+ T cells were sorted on FACSAria (BD Biosciences cell-sorter, USA) and kept at 4°C throughout sorting. Live PBMCs were gated based on forward versus side scatter profiles, followed by CD3+ based gating for T lymphocyte identification. The CD3+ positive population was subdivided by CD8/CD4 expression and used for the identification of CD8+ (CD4–, CD8+) T cells. Purity was determined for CD8+ T cells isolated by FACS for CD8+ T cells (99.8% purity). Sorted cells were immediately centrifuged and pellets were resuspended in TriPure Isolation Reagent (Roche, Germany) at -80°C until use. miRNeasy Kit (Qiagen, Germany) was used in accordance with the manufacturer protocol for the total RNA isolation from the isolated CD8+ T cells. The measurements of concentrations and purities were carried out using Nanodrop (Thermo Scientific, USA). RNA qualities were determined in Agilent Bioanalyzer (Agilent, UK) device using Agilent RNA 6000 Nano Kit.

### miRNA microarray analysis

MicroRNAs from RNA samples were marked with Cy3 fluorescent dye while transforming them into cDNA using 100ng RNA Agilent miRNA labeling Kit (Agilent, UK) and Spike Kit (Agilent, UK) and Agilent microRNA Hybridization Kit (Agilent, UK) were used to hybridize into Human miRNA Microarray, Release 19.0, 8x60K(v19) microarray slides (Agilent, UK) and scanned using Nimblegen MS200 array scanner. The TIFF image files obtained were processed using Agilent Feature Extraction software to extract raw data and obtain QC reports. The data of the samples that pass QC parameters were subject to quantile normalization and analyzed using GeneSpring GX (Agilent, UK) 13.0 software after which microRNAs with P values of < 0.05 and Fold change values of >2 and <-2 were determined to be statistically significant.

### miRNA validation with qRT-PCR analysis

For validation of miRNA expression, Universal cDNA synthesis kit (Exiqon, Denmark) was used for cDNA synthesis. Housekeeping gene (*SNORD48)* and cDNA control were controlled with spike in primers. Of the results of cDNA’s Snord48 and spike, those that are between Ct 15–29 were worked in LightCycler 480 II (Roche, Germany) using microRNA LNA^™^ primer sets (Exiqon, Denmark) and ExiLENT SYBR^®^ Green Master Mix kit for the specified miRNAs. Δ/Δ Ct method was used to carry out relative quantification for the acquired results. With this method, miRNA Ct values were normalized via snord48 and U6 house keeping genes (this value is denoted as Δ Ct) after which the groups were compared among themselves and the acquired result yielded Δ/Δ Ct. If this value is greater than 2, regulation result was determined as a Target positive expression increase and if it is less than -2, target down was determined as the result of it was controlled by the microarray data.

### KEGG Pathway Analysis of Target Genes

Pathway enrichment analysis was performed to understand the biological potency and functional classification of predicted miRNA targets. KEGG (Kyoto Encyclopedia of Genes and Genomes) [35] (http://www.genome.jp/kegg/pathway.html) pathway enrichment was used to elucidate the number of predicted miRNA target genes. WebGestalt (WEB-based GEneSeTAnaLysis Toolkit) [36] (http://bioinfo.vanderbilt.edu/webgestalt/) web based enrichment analysis tool and KEGG [35] (http://www.genome.jp/kegg/pathway.html) pathway enrichment were used to elucidate the pathways of the predicted miRNA target genes.

## Results

While 7 of the 15 patients (3 female, 4 male) were diagnosed with acute brucellosis, 8 of the patients (6 female, 2 male) were diagnosed with chronic brucellosis. The median age at diagnosis of acute brucellosis 52.14 ±10.40 and at diagnosis of chronic brucellosis was 45.00 ±11.48. In addition, the median age of 8 healthy volunteers (4 female, 4 male) were 39.62 ± 7.74.

### miRNAs play role in acute and chronic brucellosis

**Fig 1:**
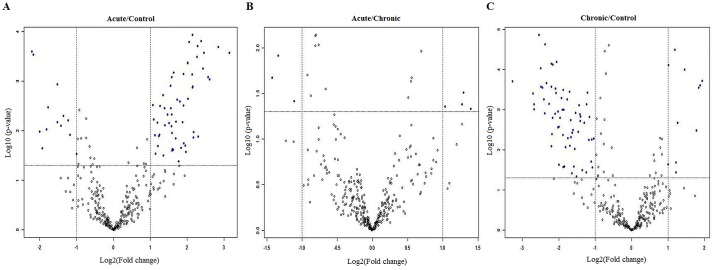
miRNAs with 2^(-Avg.(Delta(Ct)) values were evaluated between A. Acute-Control, B. Acute-Chronic C. Chronic-Control patients depend on miRNA microarray analysis (p< 0.05, cut off = 2). The volcano plot demonstrates the differential expression of the illustrated miRNAs; dots in light represent miRNAs that did not achieve significant changes in expression, dots in dark on the left indicate the miRNAswith significantly downregulated expression and dots in dark on the right indicate the miRNAswith significantly upregulated expression.

miRNA microarray data of CD8+ T cells of acute and chronic brucellosis patients and control group were analyzed. Because miRNAs with less than 2 fold alteration in expression were assumed as not significant, these miRNAs were ignored (Fig 1).

Forty-two miRNAs were altered in all brucellosis cases compared to the control group. However, the expressions of these miRNAs did not show a significant difference between chronic and acute group (Table 1).

**Table 1:** Significantly altered miRNA expressions in both chronic and acute brucellosis cases in compare to control group.

	Chronic vs control			Acute vs control			Chronic vs acute		
Symbol	Fold-change	Fold regulation	p-value*	Fold-change	Fold regulation	p-value*	Fold-change	Fold regulation	p-value*
hsa-miR-1587	10.00	10.00	0.0002	8.86	8.86	0.0003	1.13	1.13	0.5934
hsa-miR-2861	6.67	6.67	0.0004	7.17	7.17	0.0002	0.93	-1.08	0.9503
hsa-miR-3665	6.25	6.25	0.0007	6.11	6.11	0.0009	1.02	1.02	0.4252
hsa-miR-4505	5.88	5.88	0.0000	4.44	4.44	0.0001	1.32	1.32	0.0968
hsa-miR-4530	5.56	5.56	0.0003	4.45	4.45	0.0013	1.25	1.25	0.2870
hsa-miR-4507	5.56	5.56	0.0001	5.22	5.22	0.0002	1.06	1.06	0.5542
hsa-miR-1915-3p	5.56	5.56	0.0003	5.5	5.5	0.0003	1.01	1.01	0.9469
hsa-miR-4466	5.26	5.26	0.0005	5.38	5.38	0.0006	0.98	-1.02	0.9260
hsa-miR-638	5.00	5.00	0.0002	4.81	4.81	0.0003	1.04	1.04	0.7883
hsa-miR-6068	4.76	4.76	0.0007	4.41	4.41	0.0014	1.08	1.08	0.6294
hsa-miR-574-5p	4.55	4.55	0.0040	3.45	3.45	0.0509	1.32	1.32	0.5277
hsa-miR-371b-5p	4.55	4.55	0.0003	4.86	4.86	0.0002	0.94	-1.07	0.5633
hsa-miR-3162-5p	4.55	4.55	0.0081	5.93	5.93	0.0008	0.77	-1.30	0.9448
hsa-miR-4516	4.35	4.35	0.0013	3.74	3.74	0.0031	1.16	1.16	0.2854
hsa-miR-4484	4.35	4.35	0.0528	3.81	3.81	0.0818	1.14	1.14	0.5795
hsa-miR-642a-3p	4.00	4.00	0.0027	3.67	3.67	0.0067	1.09	1.09	0.6441
hsa-miR-1225-5p	4.00	4.00	0.0011	4.4	4.4	0.0007	0.91	-1.10	0.6899
hsa-miR-6090	3.70	3.70	0.0010	3.32	3.32	0.0024	1.12	1.12	0.3084
hsa-miR-4672	3.70	3.70	0.0266	4.49	4.49	0.0138	0.82	-1.21	0.4740
hsa-miR-296-5p	3.57	3.57	0.0085	3.22	3.22	0.0144	1.11	1.11	0.7435
hsa-miR-5001-5p	3.57	3.57	0.0010	3.74	3.74	0.0007	0.95	-1.05	0.8261
hsa-miR-4787-5p	3.57	3.57	0.0263	3.86	3.86	0.0200	0.93	-1.08	0.8306
hsa-miR-1234-5p	3.23	3.23	0.0079	3.02	3.02	0.0111	1.07	1.07	0.2919
hsa-miR-3960	3.13	3.13	0.0038	2.85	2.85	0.0070	1.10	1.10	0.2456
hsa-miR-3940-5p	3.13	3.13	0.0384	3.57	3.57	0.0224	0.88	-1.14	0.5633
hsa-miR-4687-3p	3.03	3.03	0.0032	2.99	2.99	0.0047	1.01	1.01	0.8516
hsa-miR-6088	3.03	3.03	0.0260	3.08	3.08	0.0239	0.98	-1.02	0.8393
hsa-miR-4281	2.94	2.94	0.0096	2.98	2.98	0.0081	0.99	-1.01	0.6237
hsa-miR-548ao-3p	2.94	2.94	0.0641	3.12	3.12	0.0747	0.94	-1.06	0.8664
hsa-miR-4687-5p	2.86	2.86	0.0466	2.63	2.63	0.0630	1.09	1.09	0.8332
hsa-miR-1273g-3p	2.78	2.78	0.0036	2.56	2.56	0.0315	1.09	1.09	0.0775
hsa-miR-6087	2.63	2.63	0.0019	2.28	2.28	0.0069	1.15	1.15	0.2146
hsa-miR-4455	2.56	2.56	0.0321	2.37	2.37	0.1439	1.08	1.08	0.8781
hsa-miR-877-3p	2.50	2.50	0.2247	2.64	2.64	0.2136	0.95	-1.06	0.6046
hsa-miR-197-3p	2.33	2.33	0.0004	3.03	3.03	0.0234	0.77	-1.30	0.2943
hsa-miR-6125	2.27	2.27	0.0056	2.18	2.18	0.0125	1.04	1.04	0.7858
hsa-miR-4312	2.08	2.08	0.0954	2.13	2.13	0.1385	0.98	-1.02	0.2967
hsa-miR-369-5p	0.48	-2.08	0.1230	0.39	-2.56	0.0050	1.23	1.23	0.0417
hsa-miR-586	0.47	-2.13	0.1222	0.47	-2.13	0.1222	1.00	1.00	0.2786
hsa-miR-520f-3p	0.43	-2.33	0.0370	0.43	-2.33	0.0910	1.00	-1.00	0.9864
hsa-miR-4307	0.41	-2.41	0.0021	0.35	-2.86	0.0012	1.19	1.19	0.5581
hsa-miR-505-3p	0.30	-3.34	0.1395	0.41	-2.44	0.1685	0.73	-1.37	0.6161

*P values were calculated using independent sample T test.

In comparison to acute cases, expression levels of 2 miRNAs were significantly altered in chronic cases. The expressions of these miRNAs were similar for acute cases and control group (Table 2).

**Table 2:** Altered miRNA expressions in chronic brucellosis.

	Chronic vs control			Acute vs control			Chronic vs acute		
Symbol	Fold-change	Fold regulation	p-value*	Fold-change	Fold regulation	p-value*	Fold-change	Fold regulation	p-value*
hsa-miR-126-5p	0.50	-2.01	0.0229	1.47	1.47	0.2583	0.34	-2.95	0.0441
hsa-miR-4753-3p	0.48	-2.10	0.2910	1.01	1.01	0.9740	0.47	-2.12	0.3036

*P values were calculated using independent sample T test.

In addition, expression levels of 5 miRNAs were significantly altered in acute cases. The expressions of these genes were similar for chronic cases and control group (Table 3).

**Table 3:** Altered miRNA expressions in acute brucellosis.

	Chronic vs control			Acute vs control			Chronic vs acute		
Symbol	Fold-change	Fold regulation	p-value*	Fold-change	Fold regulation	p-value*	Fold-change	Fold regulation	p-value*
hsa-miR-199b-5p	1.27	1.27	0.6027	2.64	2.64	0.0669	0.48	-2.09	0.0464
hsa-miR-625-3p	0.92	-1.09	0.8026	0.35	-2.86	0.0068	2.62	2.62	0.0121
hsa-miR-656-3p	0.88	-1.14	0.7855	0.37	-2.70	0.0906	2.37	2.37	0.5583
hsa-miR-6509-3p	0.86	-1.16	0.7837	2.09	2.09	0.0842	0.41	-2.42	0.0682
hsa-miR-548aq-5p	0.79	-1.27	0.4490	0.29	-3.45	0.0034	2.72	2.72	0.0211

*P values were calculated using independent sample T test.

### Predicted Target Pathways of miRNA

To understand the role of immunological and genetic factors involved in the transition of brucellosis into chronic infection, target pathway prediction of miR-126-5p andmiR-4753-3p were performed according to KEGG function annotations.

**Fig 2:**
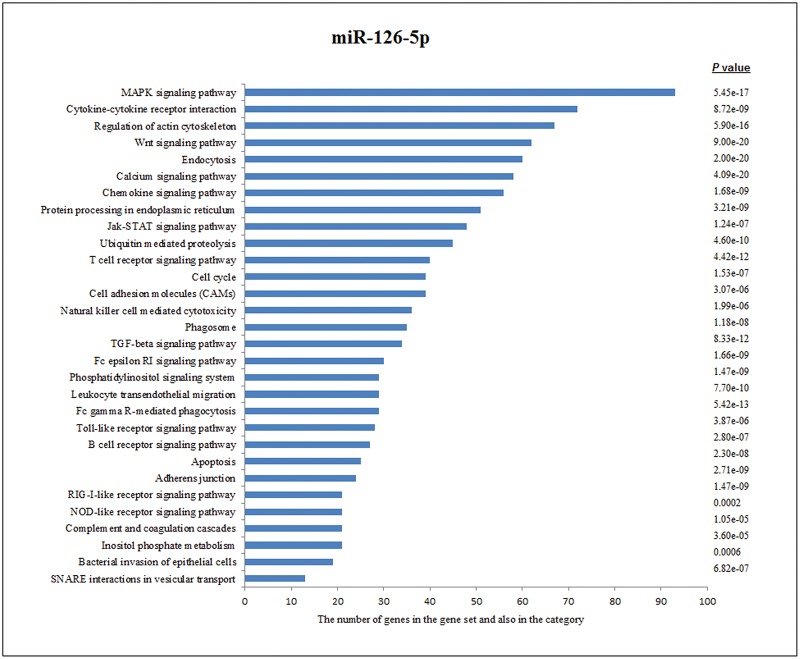
Pathway Analysis of miR-126-5p according to KEGG function annotations.

One of decreased miRNA (miR-126-5p) of target genes immunologically effective pathways are shown in Fig 2.

**Fig 3:**
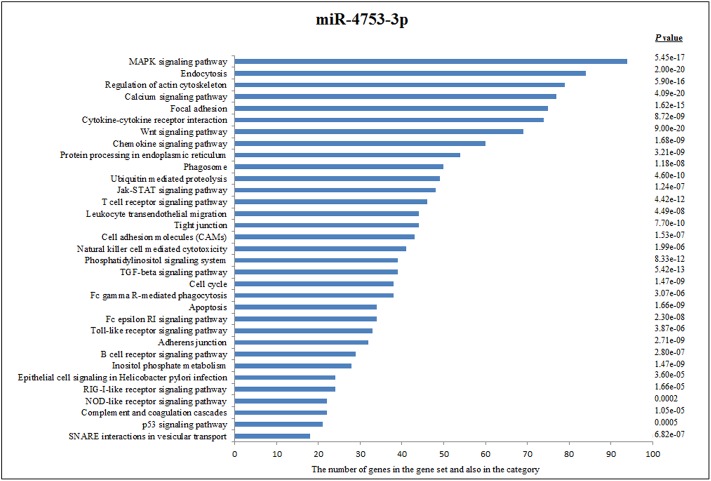
Pathway Analysis of miR-4753-3p according to KEGG function annotations.

Other decreased miRNA (miR-4753-3p) that target genes of immunologically effective pathways are shown in Fig 3.

**Fig 4:**
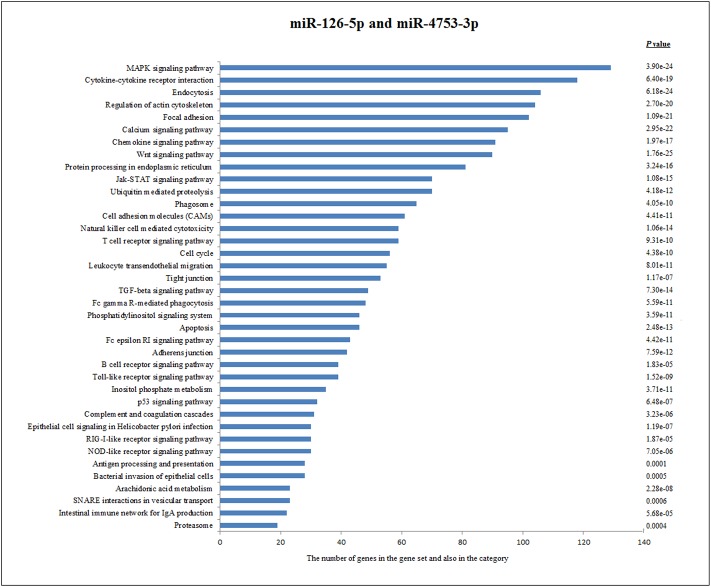
Pathway Analysis of 2 down regulated miRNAs, miR-126-5p and miR-4753-3p according to KEGG function annotations.

**Fig 5:**
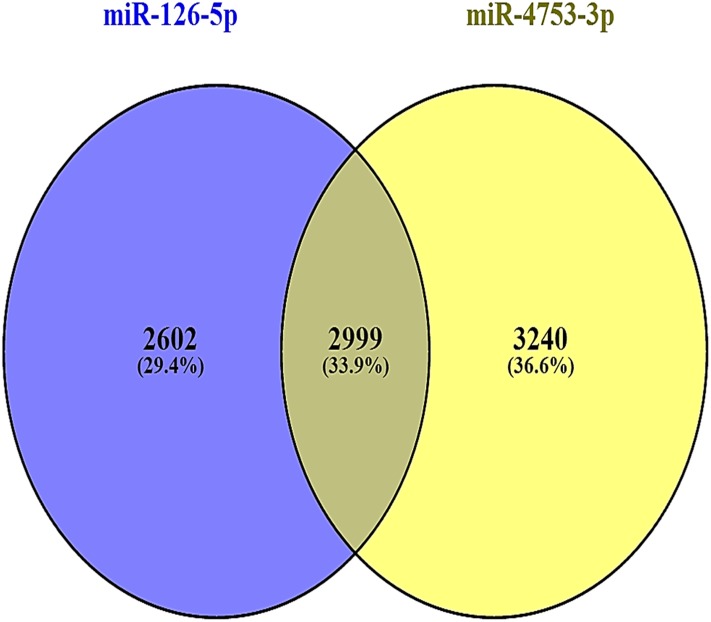
Target gene prediction for the 2 down regulated miRNAs, miR-126-5p and miR-4753-3p depend on KEGG analysis.

miRNAs (miR-126-5p and miR-4753-3p) that are down-regulated in chronic group were considered common. Results showed that about 8841 predicted genes were annotated, with the genes primarily active in MAPK signaling pathway, cytokine-cytokine receptor interactions, endocytosis, regulation of actin cytoskeleton, focal adhesion, calcium signaling pathway, chemokine signaling pathway and Wnt signaling pathway in Fig 4. The predicted number of target genes are present in Fig 5.

2999 genes (33.9%) were regulated mutually by miR-126-5p and miR-4753-3p.

According to KEGG function annotations, these mutual genes play a role in multiple signaling pathways, such as regulation of actin cytoskeleton, focal adhesion, Wnt signaling pathway, calcium signaling pathway, endocytosis, T cell receptor signaling pathway and JAK-STAT signaling pathway.

## Discussion and Conclusion

Brucella is an intracellular parasite of wordwide importance that inflicts important health problems in animals and humans. In some cases, initial infection leads to chronic and reactivating brucellosis, and causes significant morbidity and imposes a significant economic burden on healthcare systems. Our understanding of the mechanisms of the transition from acute infection to chronic brucellosis remains incomplete. Multiple host factors may be involved in this complex process. The essential role for CD8+ T cell-mediated cytotoxicity in control of brucellosis is well known. β2-microglobulin knockout mice, which cannot mount a functional CD8+ T-cell response, have significantly exacerbated brucellosis [26]. Studies using in vivo depletion of T-cell subsets have shown that CD8+ T cells are the primary responder to DNA vaccines encoding a *B*. *melitensis* and *B*. *ovis* outer membrane protein [37]. In addition, in vivo depletion of CD8+ T cells results in higher bacterial load in *B*. *abortus*-infected BALB/c mice [38]. Durward-Diioila et al. showed that chronic infection of mice with *B*. *melitensis* led to CD8+ T cell exhaustion, manifested by programmed cell death 1 (PD-1) and lymphocyte activation gene 3 (LAG-3) expression and a lack of IFN-γ production [39]. This is indicative of the importance of cytotoxic CD8+ T cells in a protective immune response against Brucella infections.

Herein, we focused on miRNAs that play key roles in the fundamental cellular processes. The expression profile of these miRNAs showed high variability between individuals and was independent of their age, gender or clinical phenotype. In this study, we investigated the expression patterns of several miRNA in CD8+ T cells of chronic, acute cases and the control group to establish a profile for the post-transcriptional regulation of gene expression and immunological basis involved in course of chronicity.

Although there have been no studies investigating miRNA profiles in brucellosis, there are few studies investigating the differences in miRNA expressions levels between acute and chronic viral infections in humans. Most of these studies are focused on circulating miRNAs in acute and chronic forms of Hepatitis or acute and chronic phases of Hepatitis C [40–42]. In our recent study, we have shown that reduced expression of miR-139-3p, miR-6069 and miR-494 and induced expression of miR-1238-3p were significantly associated with chronic brucellosis [43].

In this study, all patients received the same antibiotic regimen including doxycycline and rifampicin. In two studies, it was demonstrated that rifampicin may alter miRNA expression in human hepatocyte cultures [44, 45]. Apart from those studies, there are no studies available that display the role of antibiotics with regards to changes in miRNA expression in other cells *in vitro* or *in vivo*. Therefore, this issue was not considered in this study, since the same antibiotic regimen was used for all patients.

The findings discussed here reveal the first detailed snapshot of miRNA expression levels in PBMCs of brucellosis patients. The only study focused on miRNA expression associated with *Brucella* infections has been published by Zheng et al., which examined the miRNA expression profiles in RAW264.7 cells in response to *Brucella melitensis* infection. They reported 344 unique miRNAs which were co-expressed in the two libraries (mock- and *Brucella*-infected RAW264.7 cells) in which 57 miRNAs were differentially expressed. Eight differentially expressed miRNAs with high abundance were subjected to further analysis. The GO enrichment analysis suggests that the putative target genes of these differentially expressed miRNAs are involved in apoptosis, autophagy, and immune response. In particular, a total of 25 target genes participate in regulating apoptosis and autophagy, indicating that these miRNAs may play important regulatory roles in the *Brucella*-host interactions [46]. In our previous study, more than 2000 miRNAs were screened in peripheral blood mononuclear cells of patients with acute or chronic brucellosis and we determined that while the expression level of miR-1238-3p was increased, miR-494, miR-6069 and miR-139-3p were decreased in the chronic group in comparison to the acute infection group [43]. In this current study, the regulatory role of 2000 miRNAs were evaluated in human CD8+ T cells. We determined 42 miRNAs involved in Brucella infection and 2 miRNAs that were uniquely expressed in chronic brucellosis and 5 miRNAs uniquely expressed in acute brucellosis. None of the evaluated miRNAs were differently regulated between chronic and acute brucellosis.

### Similarly expressed miRNAs in chronic and acute Brucelosis

Forty-two miRNAs displayed similar expression trends in both the chronic and acute infection groups compared with the control group, which indicated that both chronic and acute brucellosis infections might share, at least partly, similar regulatory mechanisms. According to our findings, whereas the expression of five miRNAs (miR-369-5p, miR-586, miR-520f-3p, miR-4307 and 505-3p) were decreased, the expression of thirty-seven miRNAs increased in both the chronic and active group compared with the control group.

Among the miRNAs, the function of fifteen downregulated miRNAs (miR-1587, miR-4505, miR-1915-3p, miR-4466, miR-3162-5p, miR-4484, miR-5001-5p, miR-3940-5p, miR-4687-3p, miR-6068, miR-548ao-3p, miR-4687-5p, miR-1273g-3p, miR-877-3p, miR-4312) and the function of two miRNAs that were upregulated (miR-520f-3p, miR-4307) remain unknown.

Indeed, some miRNAs that have been previously linked to carcinogenesis of different organs and tissues, such as miR-2861 [47, 48], miR-4530 [49], miR-638 [50], miR-371b-5p [51], miR-1225-5p [52, 53], miR-296-5p [54, 55], miR-4787-5p [56], miR-4281 [57], miR-4455 [58], miR-197-3p [59], miR-369-5p [60, 61] and miR-505-3p [62] which were found to be altered in brucellosis in our analysis.

One of the 37 miRNAs where the expression was upregulated in brucellosis is miR-4516. The expression of miR-4516 was 4.35 fold and 3.74 fold in chronic group and acute infection groups respectively in comparison to the control group. Chowdhari et al. show that overexpression of hsa-miR-4516 downregulates STAT3, p-STAT3, CDK6, and UBE2N proteins that are consistently upregulated in psoriasis and induces apoptosis in HaCaT cells [63]. STAT3 mediates the expression of a variety of genes in response to cell stimuli, and thus plays a key role in many cellular processes such as cell growth and apoptosis [64]. A study conducted by Yu et al. showed that STAT3 regulates proliferation and survival of CD8+ T cells and enhances effector responses [65]. CDK6 is important for the control of G1 to S phase transition [66]. High expression of active CDK6 in the cytoplasm of CD8 memory cells favors rapid cell division [67]. The modification of proteins with ubiquitin is an important cellular mechanism for targeting abnormal or short-lived proteins for degradation. Ubiquitination involves at least three classes of enzymes: ubiquitin-activating enzymes, or E1s, ubiquitin-conjugating enzymes, or E2s, and ubiquitin-protein ligases, or E3s. *UBE2N* gene encodes a member of the E2 ubiquitin-conjugating enzyme family [68]. Thus we suggest that, miR-4516 may involve effector responses of CD8+ T cells in these patients through reducing STAT3, CDK6, and UBE2N expression.

Another set of up-regulated miRNAs in both chronic and acute brucelosis are miR-2861 and miR-3960. Li et al. showed that miR-2861 plays a positive regulatory role in osteoblast differentiation by targeting histone deacetylase 5, an enhancer of Runx2 degradation [69]. Afterwards Hu et al. identified miR-3960 that played a regulatory role in osteoblast differentiation through a regulatory feedback loop with miR-2861. They reported that miR-3960 and miR-2861, transcribed together from the same miRNA polycistron, both function in osteoblast differentiation through a novel Runx2/miR-3960/miR-2861regulatory feedback loop [70]. Xia et al. showed that Runx2/miR-3960/miR-2861 positive feedback loop plays an important role in osteogenic transdifferentiation of vascular smooth muscle cells [71]. Our findings showed that miR-2861 and miR-3960 have increased expression in both chronic and active group compared with the control group. Because all of the patients involved in this study were classified as osteoarticular brucellosis cases, our findings support the previous knowledge about the altered function of miR-2861 and miR-3960.

Increasing evidence indicates that many miRNAs which we determined significantly upregulated in both chronic and acute brucellosis are also associated with other infectious diseases. A study by Xun et al. show that the expression level of miR-4530 [72] and miR-296-5p are most differentially up-regulated in enterovirus 71 (EV71) infections [73]. Additionally, Zheng et al. showed that miR-296-5p supressed EV71 replication by targeting the viral genome [73]. In a study of Tan et al. identified miR-574-5p in serum provided high diagnostic accuracy for chronic hepatitis B with persistently normal alanine aminotransferase [74]. Asahchop et al. showed that miR-4516, which is a plasma-derived miRNA, was differentially expressed in HIV/AIDS patients with HIV-associated neurocognitive disorder [75]. There are limited studies concerning miR-3665, one of the increased miRNA in chronic and acute brucellosis patients. A study by Das et al. investigated whether *Mycobacterium tuberculosis* infection affects miRNAs of macrophages [76]. THP-1 macrophages infected with virulent (H37Rv) and avirulent (H37Ra) strains of *M*. *tuberculosis* were analyzed for changes in miRNAs' expression using miRNA microarray. This revealed that one of nine miRNA genes (miR-3665) was differentially expressed between THP-1 cells infected with *M*. *tuberculosis H37Rv* and *M*. *tuberculosis H37Ra* strains [76]. Also, miR3665 were upregulated in both acute and chronic group compare the control group in our study. This miRNA is likely to play a significant role in the pathogenesis of intracellular infection.

### Specifically altered miRNAs in Chronic Brucellosis

Describing the prognostic factors associated with chronicity of brucellosis infection is substantial for contributing to follow-up treatments and improving alternative therapy protocols for these patients afflicted with brucellosis infections. In the current study, 2 miRNAs (miR-126-5p and miR-4753-5p) were determined to display a significant fold change within chronic group in comparison with both the acute infection group and the control group. Both of miRNAs were downregulated in the chronic group compared with the acute infection group.

One of the downregulated microRNAs, miR-126-5p have been proven to be involved in the carcinogenesis and development of various types of cancer in previous studies [77–82].

MiR-126-5p is an intronic miRNA identified as tumor suppressor in many tumors. Wu et al. identified miR-126-5p was significantly downregulated in stromal cells of Giant cell tumor and affect osteoclast (OC) differentiation and bone resorption by repressing MMP-13 expression at the post-transcriptional level and osteolysis formation in GCT through negative regulation of Parathyroid hormone-related protein (PTHrP) [83, 84]. Choi et al. evaluated the roles of activated lymphocyte subsets in osteoclastogenesis and they determined that CD8+ T cells profoundly suppressed osteoclast differentiation via direct interaction [85]. In the present study the expression of miR-126-5p was decresed in CD8+ T cells. Thus, evaluation of the data shows that decreased expression of miR-126-5p in CD8 + T cells may play an important physiological role in osteoclast differentiation and osteolysis formation. Osteoarticular brucellosis is the most common presentation of the disease in humans, affecting up to 85% of patients. The three most common forms of osteoarticular involvement are sacroilitis, spondylitis, and peripheral arthritis. Loss of bone is a serious complication of localized bacterial infection of bones or the adjacent tissue. In another study, *B*. *abortus* may, directly and indirectly harm osteoblast function, contributing to the bone and joint destruction observed in patients with osteoarticular complications of brucellosis [86]. In our study, all of our cases were composed of brucellosis patients with bone and joint involvement.

According to KEGG pathways analysis, miR-126-5p was linked to MAPK signaling pathway, cytokine-cytokine receptor interaction, regulation of actin cytoskeleton, wnt signaling pathway, endocytosis, calcium signaling pathway, chemokine signaling pathway, protein processing in endoplasmic reticulum, JAK-STAT signaling pathway, ubiquitin mediated proteolysis and T cell receptor signaling pathway in chronic brucellosis pathways in chronic brucellosis patients.

The other down-regulated miRNA in chronic brucellosis was miR-4753-5p and only one study is present in press regarding its function. In this study associated with autism spectrum disorders (ASDs), it was assessed whether a brain region associated with core social impairments in ASD, the superior temporal sulcus (STS), would evidence greater transcriptional dysregulation of miRNA than adjacent, yet functionally distinct, primary auditory cortex (PAC). One of both mature miRNAs (miR-4753-5p) were differentially expressed in ASD relative to control in STS [87]. Current study uniquely demonstrated the role of altered expression of miR-4753-5p in regulation of chronic brucellosis.

According to KEGG pathways analysis, miR-4753-5p was linked to MAPK signaling pathway, endocytosis, regulation of actin cytoskeleton, calcium signaling pathway, focal adhesion, cytokine-cytokine receptor interaction, wnt signaling pathway, chemokine signaling pathways, protein processing in endoplasmic reticulum, phagosomes and ubiquitin mediated proteolysis.

In summary, KEGG pathways analysis of miR-126-5p and miR-4753-5p revealed that pathways related to these miRNAs in brucellosis have a most biological significance associated with the conversion of chronicity, including MAPK signaling pathway, cytokine-cytokine receptor interaction, endocytosis, regulation of actin cytoskeleton, focal adhesion and Wnt signaling pathways. Focal adhesion, endocytosis and regulation of actin cytoskeleton pathway is very important for intracellular bacterial clearance. Inhibition of intracellular bacterial replication is related to its control of endocytosis and membrane fusion events between endosomes and Brucella-containing phagosomes. The MAPK cascade is one of the most ancient and evolutionarily conserved signaling pathways and is involved in all aspects of immune responses in mammalian hosts. This signaling cascade is activated by different PAMPs, which play an important role in the phagocytosis of bacteria and remodeling of the actin cytoskeleton [88, 89]. Cytokines are crucial intercellular regulators and mobilizers of cells engaged in innate as well as adaptive inflammatory host defenses, cell growth, differentiation, cell death, angiogenesis, and development and repair processes aimed at the restoration of homeostasis. One of the effective signaling pathways in the secretion of cytokines is the cytokine-cytokine receptor interaction pathway. The Th1 nature of adaptive immunity in brucellosis is clearly demonstrated. The Th1 immune response against Brucella leads to IFN-γ secretion by antigen-specific T lymphocytes. One of the major producers IFN-γ is CD8+ T lymphocytes [90]. In addition, it was demonstrated that chronic infection of *Brucella* led to CD8 T cell exhaustion, manifested by programmed cell death 1 (PD-1) and lymphocyte activation gene 3 (LAG-3) expression and a lack of IFN-γ production [39]. So, JAK-STAT and T cell receptor signaling pathways which are regulated by miR-126-5p and cytokine-cytokine receptor interaction which is targeted by both miR-126-5p and miR-4753-5p may be associated with CD8 T cell exhaustion.

In our study, all of our cases were composed of brucellosis patients with bone and joint involvement. One of effective signaling pathways in the development of bone and joint was Wnt signaling pathway. Wnt signalling has been shown as an important regulatory pathway in the osteogenic differentiation of mesenchymal stem cells. Induction of the Wnt signaling pathway promotes bone formation while inactivation of the pathway leads to osteopenic states [91].

In conclusion, identifying the prognostic factors for chronicity is required for follow-up treatments and improving alternative therapy protocols in brucellosis infection. In the present study we uniquely determined that reduced expression of miR-126-5p and miR-4753-5p were significantly associated with chronic brucellosis in human CD8+ T cells. The predicted target genes of these miRNAs are involved in MAPK signaling pathway, cytokine-cytokine receptor interactions, endocytosis, regulation of actin cytoskeleton, focal adhesion and Wnt signaling pathway indicating their potential roles in chronic brucellosis and progress. Further research and validations are needed to evaluate the potential target genes of these miRNAs and their relation to two miRNAs discussed in this study may be regulator miRNAs of novel candidate biomarker genes indicating progression to chronicity of brucellosis in CD8+ T cells.
